# The appropriateness of Bland-Altman’s approximate confidence intervals for limits of agreement

**DOI:** 10.1186/s12874-018-0505-y

**Published:** 2018-05-22

**Authors:** Gwowen Shieh

**Affiliations:** Department of Management Science, National Chiao Tung University, Hsinchu, 30010 Taiwan, Republic of China

**Keywords:** Assurance probability, Expected width, Precision, Quantile, Sample size

## Abstract

**Background:**

Percentiles are widely used as reference limits for determining the relative magnitude and substantial importance of quantitative measurements. An important application is the advocated Bland-Altman limits of agreement.

**Methods:**

To contribute to the data analysis and design planning of reference limit or percentile research, the purpose of this paper is twofold. The first is to clarify the statistical features of interval estimation procedures for normal percentiles. The second goal is to provide sample size procedures for precise interval estimation of normal percentiles.

**Results:**

The delineation demonstrates the theoretical connections between different pivotal quantities for obtaining exact confidence intervals. Moreover, the seemingly accurate approximate methods with equidistant from the principal estimators are shown to have undesirable confidence limits. It is found that the optimal sample size has a minimum for median or mean, and increases as the percentile approaches the extremes.

**Conclusions:**

The exact interval procedure should be used in preference to the approximate methods. Computer algorithms are presented to implement the suggested interval precision and sample size calculations for planning percentile research.

**Electronic supplementary material:**

The online version of this article (10.1186/s12874-018-0505-y) contains supplementary material, which is available to authorized users.

## Background

A percentile is a numerical measure that represents the reference point below which a given percentage of values in the target population fall. Because of the conceptual simplicity and context-free feature, percentiles are widely used for determining the relative magnitude and substantial importance of quantitative measurements in all scientific fields. For example, the children health conditions are often assessed by their weight and height in comparison to the national averages and percentiles found in the growth charts. Also, reference limits are extensively applied in medicine and related fields to identify informative range of measurement from a reference population. The most typical reference limits contain the central 95% of the values in the population of interest. As an important application, the Bland and Altman [1, 2] 95% limits of agreement are comprised of the 2.5th percentile and 97.5th percentile for the distribution of the difference between paired measurements.

The practical usage of percentiles is often represented by referring to a normal distribution. In this prominent case, the normal percentile is a linear function of the mean and standard deviation of the designated population. Note that the sample mean and sample variance are complete and sufficient statistics for the population mean and variance. Although estimation of normal percentile is not discussed in most standard texts, it is straightforward to obtain the minimum variance unbiased estimator of a normal percentile. However, the dominance property does not extend to other principles in decision theoretic analyses such as the mean square error criterion. Among others, Royston and Mathews [3] conducted a comparison of potential point estimators of normal percentiles with respect to bias and mean square error. More advanced and theoretical investigations of normal percentile estimators can be found in Keating, Mason, and Balakrishnan [4], Keating and Tripathi [5], Parrish [6], Rukhin [7], and Zidek [8, 9].

In view of the stochastic nature in statistical inference, it is more informative to construct confidence intervals for the target parameters than to provide a single estimate about their values. General expositions and comprehensive guidelines of interval estimation are available in Hahn [10, 11], Hahn and Meeker [12], and Vardeman [13]. Accordingly, various interval methods of normal percentiles have been described from different perspectives. The exact interval procedure of normal percentiles has been documented in the literature, for example, see Hahn and Meeker [12], Johnson, Kotz, and Balakrishnan [14], and Owen [15]. Moreover, the one-sided confidence intervals of normal percentiles have a close link to the one-sided tolerance bounds of a normal distribution as noted in David and Nagaraja [16], Krishnamoorthy and Mathew [17], and Odeh and Owen [18].

Notably, Bland and Altman [1, 2] suggested the 95% limits of agreement for evaluating the differences between measurements by two methods. The endpoints of the Bland-Altman 95% limits of agreement are the 2.5th percentile and 97.5th percentile for the distribution of the difference between paired measurements. To reflect the uncertainty due to sampling error, approximate interval formulas were presented for estimating the two individual percentiles. The large number of citations revealed that the Bland-Altman analysis has become the major technique for assessing agreement between two methods of clinical measurement. But the recent work of Carkeet [19] and Carkeet and Goh [20] provided detailed discussions in favor of exact confidence interval over the approximate procedure considered in Bland and Altman [1, 2], especially when the sample sizes are small. Further considerations and reviews of measuring agreement in method comparison studies are available in Barnhart, Haber, and Lin [21], Choudhary and Nagaraja [22], and Lin et al. [23].

Although the practical implementation of the exact interval procedure is well presented in Carkeet [19], the explication of the differences between the exact and approximate methods mainly concentrated on the relative magnitudes and symmetric/asymmetric bounds of the resulting confidence limits. On the other hand, the endpoints of the Bland-Altman 95% limits of agreement are usually viewed as a pair of bound for measuring agreement in method comparison studies. Accordingly, Carkeet [19] and Carkeet and Goh [20] focused on the comparison of the approximate confidence intervals for upper and lower limits of agreements as a pair and the exact two-sided tolerance intervals for a normal distribution. Therefore, the distinctive advantage of the exact interval procedures and the potential limitation of the approximate confidence intervals for the individual upper and lower limits of agreement were not fully addressed in Carkeet [19] and Carkeet and Goh [20]. It is of practical importance to conduct a detailed appraisal of the accuracy and discrepancy between the exact and approximate interval procedures for an individual limit of agreement under a wide range of model configurations. The problem of obtaining a single confidence interval to cover both limits of agreement simultaneously is more involved and a detailed discussion of this topic is beyond the scope of the present study.

In addition to the abovementioned studies, a numerical comparison of several interval estimation methods of normal percentiles was presented in Chakraborti and Li [24]. They adopted a standardized minimum variance unbiased estimator as the pivotal quantity and proposed both exact and approximate confidence intervals of normal percentiles. Their simulation study showed that the expected width and coverage probability of the suggested exact and approximate methods are nearly identical to that of the procedure described in Lawless ([25], p. 231). Despite the analytic arguments and empirical findings in Chakraborti and Li [24], the following two attentions toward their illustration should be noted. First, although it was demonstrated that Lawless’s [25] confidence intervals are the same as the existing formulas in Owen [15] and Odeh and Owen [18], they did not discuss the theoretical implications between their exact method and the established exact procedure. Second, in contrast to the asymmetry of the exact confidence intervals, the approximate confidence intervals of Chakraborti and Li [24] are equidistant around the minimum variance unbiased estimate. Note that the two endpoints of a two-sided confidence interval can also be interpreted as the limits of one-sided confidence interval. Thus, the performance of the two limits of Chakraborti and Li’s [24] approximate interval method should be further evaluated with respect to the equal-tailed property. The analytic and numerical results in Chakraborti and Li [24] are not detailed enough to clarify these fundamental issues. It is prudent to elucidate these vital aspects of their methods to be accepted as a feasible technique.

To enhance the adoption of appropriate techniques for interval estimation and research design, this paper has two objectives. The first is to appraise the statistical features of interval estimation procedures for normal percentiles. Theoretical justifications are presented to illuminate the statistical connections between different pivotal quantities for obtaining exact confidence intervals. Furthermore, comprehensive empirical assessments are provided to show the seemingly accurate approximate methods with equidistant around the principal estimators have problematic confidence limits. The second goal is to provide sample size procedures for precise interval estimation of normal percentiles. The required precision of a confidence interval is evaluated with the magnitude of expected width, and the assurance probability of interval width within a designated threshold. In view of the general availability of statistical software packages SAS and R, computer algorithms are developed to facilitate the implementation of the suggested confidence interval and sample size computations.

## Methods

Assume *X*_1_, …, *X*_*N*_ are a sample from a *N*(μ, σ^2^) population with unknown mean μ and variance σ^2^ for *N* > 1. The sample mean $\overline{X}$ and sample variance *S*^2^ are defined as $\overline{X}=\sum \limits_{i=1}^N{X}_i/N$ and ${S}^2=\sum \limits_{i=1}^N{\left({X}_i-\overline{X}\right)}^2/\left(N-1\right)$, respectively. The 100*p*th percentile of the distribution *N*(μ, σ^2^) is denoted by θ, where

1 $$ \uptheta =\upmu +{z}_p\upsigma $$

and *z*_*p*_ is the 100*p*th percentile of the standard normal distribution *N*(0, 1). To estimate the percentile θ, the intuitive formula

2 $$ {\widehat{\uptheta}}_B=\overline{X}+{z}_pS $$

is a biased estimator because *E*[*S*] < σ. As noted in Royston and Mathews [3], the minimum variance unbiased estimator is

3 $$ {\widehat{\uptheta}}_{MU}=\overline{X}+{z}_p cS. $$

where *c* = (ν/2)^1/2^Γ(ν/2)/Γ{(ν + 1)/2} and ν = *N* – 1. Note that *c* is an adjusting factor so that *cS* is an unbiased estimator of σ or *E*[*cS*] = σ. Moreover, it can be shown that the variance and mean square error of the two estimators are *Var*[$\widehat{\uptheta}$_*B*_] = {1 + *N*${z}_p^2$(1–1/*c*^2^)}(σ^2^/*N*), *MSE*[$\widehat{\uptheta}$_*B*_] = {1 + 2*N*${z}_p^2$(1–1/*c*)}(σ^2^/*N*), and *Var*[$\widehat{\uptheta}$_*MU*_] = *MSE*[$\widehat{\uptheta}$_*MU*_] = {1 + *N*${z}_p^2$(*c*^2^–1)}(σ^2^/*N*). Because *c* is slightly larger than 1 for *N* > 1, further examinations assure the contrasting dominance phenomena: *Var*[$\widehat{\uptheta}$_*MU*_] > *Var*[$\widehat{\uptheta}$_*B*_] and *MSE*[$\widehat{\uptheta}$_*MU*_] > *MSE*[$\widehat{\uptheta}$_*B*_]. The relative numerical performance of $\widehat{\uptheta}$_*B*_, $\widehat{\uptheta}$_*MU*_, and alternative estimators of θ can also be found in Royston and Mathews [3].

To obtain confidence intervals for θ, standard derivations show that

4 $$ {T}^{\ast }=\frac{\overline{X}-\uptheta}{S/{N}^{1/2}}\sim t\left(v,-{z}_p{N}^{1/2}\right), $$

where *t*(ν, –*z*_*p*_*N*^1/2^) is a noncentral *t* distribution with degrees of freedom ν and noncentrality parameter –*z*_*p*_*N*^1/2^ (Johnson, Kotz, & Balakrishnan [14], Chapter 31). Accordingly, T* yields a pivotal quantity for constructing confidence intervals of normal percentiles. An upper 100(1 – α)% one-sided confidence interval of θ is expressed as {$\widehat{\uptheta}$_*L*_, ∞} and the lower confidence limit is

5 $$ {\widehat{\uptheta}}_L=\overline{X}-{t}_{1-\upalpha}\left(v,-{z}_p{N}^{1/2}\right)S/{N}^{1/2}=\overline{X}+{t}_{\upalpha}\left(v,{z}_p{N}^{1/2}\right)S/{N}^{1/2}, $$

where *t*_1 − α_(ν, –*z*_*p*_*N*^1/2^) is the 100(1 – α)th percentile of the distribution *t*(ν, –*z*_*p*_*N*^1/2^) and *t*_1 − α_(ν, –*z*_*p*_*N*^1/2^) = −*t*_α_(ν, *z*_*p*_*N*^1/2^) for 0 < α < 1. Also, a lower 100(1 – α)% one-sided confidence interval of θ is {−∞, $\widehat{\uptheta}$_*U*_} and the upper confidence limit has the form

6 $$ {\widehat{\uptheta}}_U=\overline{X}-{t}_{\upalpha}\left(v,-{z}_p{N}^{1/2}\right)S/{N}^{1/2}=\overline{X}+{t}_{1-\upalpha}\left(v,{z}_p{N}^{1/2}\right)S/{N}^{1/2}. $$

Furthermore, a 100(1 – α)% two-sided confidence interval of θ with equal tail probability can be readily obtained as {$\widehat{\uptheta}$_*L*_, $\widehat{\uptheta}$_*U*_} where

$$ {\widehat{\uptheta}}_L=\overline{X}-{t}_{1-\upalpha /2}\left(v,-{z}_p{N}^{1/2}\right)S/{N}^{1/2}=\overline{X}+{t}_{\upalpha /2}\left(v,{z}_p{N}^{1/2}\right)S/{N}^{1/2} $$

and

7 $$ {\widehat{\uptheta}}_U=\overline{X}-{t}_{\upalpha /2}\left(v,-{z}_p{N}^{1/2}\right)S/{N}^{1/2}=\overline{X}+{t}_{1-\upalpha /2}\left(v,{z}_p{N}^{1/2}\right)S/{N}^{1/2}. $$

Supplementary SAS/IML and R computer programs are provided to take advantage of the embedded statistical functions for calculating the exact confidence intervals.

In addition, it may be more appealing to modify the point estimators $\widehat{\uptheta}$_*B*_ and $\widehat{\uptheta}$_*MU*_ to acquire the alternative pivotal quantities

8 $$ {T}_B=\frac{{\widehat{\uptheta}}_B-\uptheta}{S/{N}^{1/2}}\ \mathrm{and}\ {T}_{MU}=\frac{{\widehat{\uptheta}}_{MU}-\uptheta}{S/{N}^{1/2}} $$

for deriving the confidence intervals of θ, respectively. It is easy to see that *T*_*B*_ = T* + *z*_*p*_*N*^1/2^ and *T*_*MU*_ = T* + *z*_*p*_*cN*^1/2^. Therefore, *T*_*B*_ and *T*_*MU*_ differ from T* only in the location shift. Because the terms *z*_*p*_*N*^1/2^ and *z*_*p*_*cN*^1/2^ do not depend on the unknown parameters, *T*_*B*_ and *T*_*MU*_ give the same one- and two-sided confidence intervals for θ described in Eqs. 5–7. As a generalization of the simple location shifts between different pivotal quantities, the prescribed application of pivotal quantity for exact interval estimation extends to any linear function of T*. For example, Lawless [25] constructed the confidence intervals of normal percentiles through the quantity

9 $$ {T}_L=\frac{{\widehat{\uptheta}}_B-\uptheta}{S}. $$

Evidently, *T*_*L*_ can be expressed as a linear transformation of T* by *T*_*L*_ = (T* + *z*_*p*_*N*^1/2^)/*N*^1/2^. Assume *q*_*L*, 1 − α_ is the 100(1 – α)th percentile of *T*_*L*_, it is readily established that *q*_*L*, 1 − α_ = {*t*_1 − α_(*v*, −*z*_*p*_*N*^1/2^) + *z*_*p*_*N*^1/2^}/*N*^1/2^. Although the result in Lawless ([25], p. 231) is written in a different form, the quantity *T*_*L*_ also leads to the same exact confidence interval {$\widehat{\uptheta}$_*L*_, $\widehat{\uptheta}$_*U*_} for θ.

On the other hand, Chakraborti and Li [24] considered the standardized quantity

10 $$ {T}_{ST}=\frac{{\widehat{\uptheta}}_{MU}-\uptheta}{a^{1/2}S/{N}^{1/2}} $$

for interval estimation of θ, where *a* = 1 + *N*${z}_p^2$(*c*^2^–1). Their method relies on direct computations with the derived probability density function and cumulative distribution function of *T*_*ST*_. Therefore, a special purpose algorithm is required to compute the quantiles of *T*_*ST*_ and to obtain the suggested confidence intervals of θ. Note that *T*_*ST*_ is a linear function of T* in terms of *T*_*ST*_ = (T* + *z*_*p*_*cN*^1/2^)/*a*^1/2^. Hence, if *q*_*ST*, 1 − α_ denotes the 100(1 – α)th percentile of *T*_*ST*_, it has the identical linear transform with the 100(1 – α)th percentile of T* or *q*_*ST*, 1 − α_ = {*t*_1 − α_(*v*, −*z*_*p*_*N*^1/2^) + *z*_*p*_*cN*^1/2^}/*a*^1/2^. As noted earlier, the actual value *t*_1 − α_(ν, –*z*_*p*_*N*^1/2^) can be obtained with the cumulative distribution function of a noncentral *t* distribution in major statistical packages such as SAS and R. Hence with the general availability of software systems and the underlying linear relationship between *T*_*ST*_ and T*, direct calculation is not required to compute the percentile *q*_*ST*, 1 − α_. More importantly, using the standard pivotal procedure and the prescribed linear transformation of T*, the pivotal quantity *T*_*ST*_ leads to the same interval estimators of θ with T* and the other three pivotal measures *T*_*B*_, *T*_*MU*_, and *T*_*L*_. Although the pivotal quantity *T*_*L*_ was also examined in Chakraborti and Li [24], the resulting interval estimators of *T*_*L*_ and *T*_*ST*_ are viewed as two distinct procedures. However, the numerical assessments in Chakraborti and Li [24] reported that the performances of the two interval procedures of *T*_*L*_ and *T*_*ST*_ are almost identical. The important connections between the pivotal quantities and the resulting confidence intervals of θ should be properly recognized. Essentially, the prescribed explication illuminates the conceptual equivalence between the five pivotal quantities T^*^, *T*_*B*_, *T*_*MU*_, *T*_*L*_, and *T*_*ST*_ for constructing confidence intervals of θ.

## Results

Along with the exact confidence interval procedure of normal percentiles, Chakraborti and Li [24] also described an approximate interval estimator by assuming *T*_*ST*_ has a *t* distribution with degrees of freedom ν:

11 $$ {T}_{ST}\dot{\sim}t(v). $$

Thus, an approximate 100(1 – α)% two-sided equal tail confidence interval {$\widehat{\uptheta}$_*AL*_, $\widehat{\uptheta}$_*AU*_} of θ is immediately constructed as

$$ {\widehat{\uptheta}}_{AL}={\widehat{\uptheta}}_{MU}-{t}_{1-\upalpha /2}(v){a}^{1/2}S/{N}^{1/2}=\overline{X}+{\uptau}_{AL}S/{N}^{1/2} $$

and

12 $$ {\widehat{\uptheta}}_{AU}={\widehat{\uptheta}}_{MU}+{t}_{1-\upalpha /2}(v){a}^{1/2}S/{N}^{1/2}=\overline{X}+{\uptau}_{AU}S/{N}^{1/2}, $$

where τ_*AL*_ = *z*_*p*_*cN*^1/2^ – *t*_1 − α/2_(ν)*a*^1/2^, τ_*AU*_ = *z*_*p*_*cN*^1/2^ + *t*_1 − α/2_(ν)*a*^1/2^, and *t*_1 − α/2_(ν) is the 100(1 – α/2)th percentile of the distribution *t*(ν). Although the two-sided confidence interval is only an approximation, the simulation study of Chakraborti and Li [24] revealed that {$\widehat{\uptheta}$_*AL*_, $\widehat{\uptheta}$_*AU*_} is very competitive with the exact interval estimator {$\widehat{\uptheta}$_*L*_, $\widehat{\uptheta}$_*U*_} with respect to the coverage probability and interval width.

On the other hand, to construct confidence intervals of limits of agreement or percentiles, Bland and Altman [2] argued that *Var*[*S*] ≐ σ^2^/(2ν) and *Var*[$\widehat{\uptheta}$_*B*_] ≐ *b*σ^2^/*N* where $b=1+{z}_p^2/2$. With the approximation, they suggested the simplified pivotal quantity

13 $$ {T}_{BA}=\frac{{\widehat{\uptheta}}_B-\uptheta}{b^{1/2}S/{N}^{1/2}}\dot{\sim}t(v). $$

Accordingly, the widely used confidence intervals of Bland and Altman [2] can be derived from *T*_*BA*_ and they are written as {$\widehat{\uptheta}$_*BAL*_, $\widehat{\uptheta}$_*BAU*_} where

$$ {\widehat{\uptheta}}_{BAL}={\widehat{\uptheta}}_B-{t}_{1-\upalpha /2}(v){b}^{1/2}S/{N}^{1/2}=\overline{X}+{\uptau}_{BAL}S/{N}^{1/2} $$

and

14 $$ {\widehat{\uptheta}}_{BAU}={\widehat{\uptheta}}_B+{t}_{1-\upalpha /2}(v){b}^{1/2}S/{N}^{1/2}=\overline{X}+{\uptau}_{BAU}S/{N}^{1/2}, $$

with τ_*BAL*_ = *z*_*p*_*N*^1/2^ – *t*_1 − α/2_(ν)*b*^1/2^ and τ_*BAU*_ = *z*_*p*_*N*^1/2^ + *t*_1 − α/2_(ν)*b*^1/2^. For the particular case of α = 0.05, the general expressions reduce to the confidence intervals for the two endpoints of the 95% limits of agreement considered in Bland and Altman [2]:

15 $$ \overline{X}-(1.96)S\pm {t}_{0.975}(v){(2.92)}^{1/2}S/{N}^{1/2} $$

and

16 $$ \overline{X}+(1.96)S\pm {t}_{0.975}(v){(2.92)}^{1/2}S/{N}^{1/2}, $$

respectively, because *z*_0.025_ = − 1.96, *z*_0.975_ = 1.96, and *b* = 2.92.

For the blood pressure data presented in Bland and Altman [2] with the sample size *N* = 85, the sample mean difference (observer minus machine) $\overline{X}$ = − 16.29 mmHg, and the standard deviation of the differences *S* = 19.61, the 95% confidence intervals of the exact and two approximate methods for the 2.5th percentile are {$\widehat{\uptheta}$_*L*_, $\widehat{\uptheta}$_*U*_} = {− 62.9501, − 48.3770}, {$\widehat{\uptheta}$_*AL*_, $\widehat{\uptheta}$_*AU*_} = {− 62.1035, − 47.5754}, and {$\widehat{\uptheta}$_*BAL*_ and $\widehat{\uptheta}$_*BAU*_} = {− 61.9536, − 47.4961}, respectively. For the interval estimation of the 97.5th percentile, the resulting exact and two approximate 95% confidence intervals are {$\widehat{\uptheta}$_*L*_, $\widehat{\uptheta}$_*U*_} = {15.7970, 30.3701}, {$\widehat{\uptheta}$_*AL*_, $\widehat{\uptheta}$_*AU*_} = {14.9954, 29.5235}, and {$\widehat{\uptheta}$_*BAL*_, $\widehat{\uptheta}$_*BAU*_} = {14.9161, 29.3736}, respectively. Although the differences between these estimates may not be substantial, it is vital to point out that the confidence limits of the 2.5th percentile are in the ascending order of $\widehat{\uptheta}$_*L*_ < $\widehat{\uptheta}$_*AL*_ < $\widehat{\uptheta}$_*BAL*_ and $\widehat{\uptheta}$_*U*_ < $\widehat{\uptheta}$_*AU*_ < $\widehat{\uptheta}$_*BAU*_. Whereas the confidence limits of the 97.5th percentile have a reversed situation: $\widehat{\uptheta}$_*BAL*_ < $\widehat{\uptheta}$_*AL*_ < $\widehat{\uptheta}$_*L*_ and $\widehat{\uptheta}$_*BAU*_ < $\widehat{\uptheta}$_*AU*_ < $\widehat{\uptheta}$_*U*_. This inherent relationship between the three interval procedures is further justified as the usual occurrence in the simulation study.

In general, the actual distribution of the pivotal quantity T* is skewed, especially when sample size is small and *p* deviates considerably from 0.5. This implies that the interval procedure should adopt asymmetric confidence intervals for θ. Notably, the exact two-sided interval estimates {$\widehat{\uptheta}$_*L*_, $\widehat{\uptheta}$_*U*_} are not equidistant from the sample mean except for the special case *p* = 0.5. In contrast, the approximate confidence intervals {$\widehat{\uptheta}$_*AL*_, $\widehat{\uptheta}$_*AU*_} of Chakraborti and Li [24] is equidistant about the unbiased estimate $\widehat{\uptheta}$_*UB*_. Therefore, the interval procedure is presumably inappropriate and the two confidence limits $\widehat{\uptheta}$_*AL*_ and $\widehat{\uptheta}$_*AU*_ are methodologically inaccurate when one-sided coverage probabilities are considered. But the numerical investigations in Chakraborti and Li [24] did not cover these fundamental issues. Similarly, the confidence intervals {$\widehat{\uptheta}$_*BAL*_, $\widehat{\uptheta}$_*BAU*_} of Bland and Altman [2] are symmetric around the estimate $\widehat{\uptheta}$_*B*_ and thus also suffer the same shortcoming as the intervals {$\widehat{\uptheta}$_*AL*_, $\widehat{\uptheta}$_*AU*_} of Chakraborti and Li [24].

Note that the lower and upper confidence limits of a 100(1 – α)% two-sided confidence interval are equivalent to the lower and upper confidence limits of the 100(1 – α/2)% one-sided upper and lower confidence intervals, respectively. To demonstrate the potential drawback of the approximate interval procedures of Chakraborti and Li [24] and Bland and Altman [2], a simulation study was conducted to evaluate the coverage performance of their one- and two-sided confidence intervals. Although the approximate interval method of Bland and Altman [2] has been examined in Carkeet and Goh [20] under a different perspective, the particular method is included in the following appraisal for the sake of completeness and with the intention to explicate additional properties that were not reported before.

Specifically, Monte Carlo simulation studies of 10,000 iterations were performed to compute the simulated coverage probability of the exact and approximate confidence intervals for the percentiles of a standard normal distribution *N*(0, 1). The designated sample size has six different magnitudes: *N* = 10, 20, 30, 50, 100, and 200. Also, a total of eight percentile probabilities are examined: *p* = 0.025, 0.05, 0.10, 0.20, 0.80, 0.90, 0.95, and 0.975. For each replicate, the lower and upper confidence limits {$\widehat{\uptheta}$_*L*_, $\widehat{\uptheta}$_*U*_}, {$\widehat{\uptheta}$_*AL*_, $\widehat{\uptheta}$_*AU*_}, and {$\widehat{\uptheta}$_*BAL*_, $\widehat{\uptheta}$_*BAU*_} were computed to construct the 95 and 97.5% one-sided confidence intervals and the corresponding 90 and 95% two-sided confidence intervals. The simulated coverage probability was the proportion of the 10,000 replicates whose confidence interval contained the population normal percentile. Then, the adequacy of the one- and two-sided interval procedures is determined by the error = simulated coverage probability – nominal coverage probability. The results are summarized in Tables 1, 2, 3 and 4 for the exact and approximate confidence intervals with two-sided confidence coefficient 1 – α = 0.90 and 0.95, respectively.

**Table 1:** The error between simulated coverage probability and nominal coverage probability for the 90% two-sided and 95% one-sided confidence intervals when *N* = 10, 20, and 30

		Exact approach			Chakraborti and Li [24]			Bland and Altman [2]		
*N*	*p*	Upper	Lower	Two-sided	Upper	Lower	Two-sided	Upper	Lower	Two-sided
		95% CI	95% CI	90% CI	95% CI	95% CI	90% CI	95% CI	95% CI	90% CI
10	0.025	− 0.0003	0.0013	0.0010	− 0.0418	0.0447	0.0029	− 0.0604	0.0455	− 0.0149
	0.05	0.0014	0.0011	0.0025	− 0.0396	0.0436	0.0040	− 0.0541	0.0442	− 0.0099
	0.10	− 0.0005	0.0024	0.0019	−0.0368	0.0407	0.0039	− 0.0511	0.0418	− 0.0093
	0.20	0.0012	−0.0024	−0.0012	− 0.0269	0.0313	0.0044	−0.0361	0.0328	−0.0033
	0.80	0.0021	0.0006	0.0027	0.0303	−0.0282	0.0021	0.0319	−0.0370	− 0.0051
	0.90	0.0013	0.0036	0.0049	0.0392	−0.0384	0.0008	0.0404	−0.0519	− 0.0115
	0.95	0.0017	0.0001	0.0018	0.0428	−0.0414	0.0014	0.0435	−0.0593	− 0.0158
	0.975	−0.0048	0.0031	−0.0017	0.0434	−0.0435	− 0.0001	0.0440	− 0.0617	− 0.0177
20	0.025	0.0041	0.0041	0.0082	−0.0274	0.0331	0.0057	−0.0398	0.0348	−0.0050
	0.05	0.0035	0.0029	0.0064	−0.0254	0.0327	0.0073	−0.0362	0.0342	−0.0020
	0.10	−0.0018	−0.0030	− 0.0048	− 0.0257	0.0272	0.0015	− 0.0364	0.0292	− 0.0072
	0.20	0.0005	− 0.0015	− 0.0010	− 0.0217	0.0228	0.0011	−0.0276	0.0244	−0.0032
	0.80	0.0021	0.0015	0.0036	0.0241	−0.0201	0.0040	0.0256	−0.0257	− 0.0001
	0.90	0.0019	−0.0022	− 0.0003	0.0314	−0.0282	0.0032	0.0329	−0.0382	− 0.0053
	0.95	0.0027	− 0.0007	0.0020	0.0345	−0.0324	0.0021	0.0364	−0.0415	− 0.0051
	0.975	− 0.0055	− 0.0027	− 0.0082	0.0323	− 0.0347	− 0.0024	0.0342	−0.0467	− 0.0125
30	0.025	0.0014	−0.0008	0.0006	−0.0210	0.0262	0.0052	−0.0285	0.0278	−0.0007
	0.05	0.0010	−0.0027	− 0.0017	− 0.0208	0.0238	0.0030	− 0.0271	0.0263	− 0.0008
	0.10	−0.0035	0.0016	−0.0019	− 0.0261	0.0255	−0.0006	− 0.0341	0.0272	− 0.0069
	0.20	0.0007	−0.0040	− 0.0033	− 0.0184	0.0152	− 0.0032	− 0.0231	0.0174	− 0.0057
	0.80	0.0019	− 0.0046	−0.0027	0.0208	−0.0216	− 0.0008	0.0225	− 0.0253	− 0.0028
	0.90	0.0028	−0.0033	−0.0005	0.0259	−0.0256	0.0003	0.0277	−0.0319	−0.0042
	0.95	0.0031	0.0030	0.0061	0.0264	−0.0204	0.0060	0.0289	−0.0284	0.0005
	0.975	0.0019	0.0003	0.0022	0.0268	−0.0260	0.0008	0.0291	−0.0339	−0.0048

**Table 2:** The error between simulated coverage probability and nominal coverage probability for the 90% two-sided and 95% one-sided confidence intervals when *N* = 50, 100, and 200

		Exact approach			Chakraborti and Li [24]			Bland and Altman [2]		
*N*	*p*	Upper	Lower	Two-sided	Upper	Lower	Two-sided	Upper	Lower	Two-sided
		95% CI	95% CI	90% CI	95% CI	95% CI	90% CI	95% CI	95% CI	90% CI
50	0.025	− 0.0010	− 0.0026	− 0.0036	− 0.0185	0.0193	0.0008	− 0.0237	0.0214	− 0.0023
	0.05	−0.0017	− 0.0031	− 0.0048	− 0.0193	0.0172	− 0.0021	− 0.0238	0.0197	− 0.0041
	0.10	− 0.0007	− 0.0033	− 0.0040	− 0.0165	0.0158	− 0.0007	− 0.0217	0.0179	− 0.0038
	0.20	0.0022	−0.0020	0.0002	− 0.0124	0.0136	0.0012	− 0.0156	0.0161	0.0005
	0.80	0.0006	−0.0006	0.0000	0.0160	− 0.0140	0.0020	0.0179	−0.0173	0.0006
	0.90	0.0002	−0.0015	− 0.0013	0.0198	−0.0183	0.0015	0.0225	−0.0229	−0.0004
	0.95	0.0006	0.0013	0.0019	0.0221	−0.0179	0.0042	0.0246	−0.0222	0.0024
	0.975	0.0013	−0.0025	−0.0012	0.0221	−0.0233	− 0.0012	0.0254	− 0.0299	− 0.0045
100	0.025	0.0027	−0.0012	0.0015	−0.0105	0.0137	0.0032	−0.0138	0.0157	0.0019
	0.05	0.0038	−0.0009	0.0029	−0.0116	0.0112	−0.0004	−0.0152	0.0135	−0.0017
	0.10	−0.0017	−0.0026	− 0.0043	− 0.0153	0.0120	−0.0033	− 0.0182	0.0132	− 0.0050
	0.20	−0.0018	0.0035	0.0017	−0.0116	0.0134	0.0018	−0.0130	0.0147	0.0017
	0.80	0.0050	0.0007	0.0057	0.0126	−0.0094	0.0032	0.0145	− 0.0114	0.0031
	0.90	0.0016	0.0007	0.0023	0.0141	−0.0093	0.0048	0.0158	−0.0124	0.0034
	0.95	0.0028	−0.0033	− 0.0005	0.0162	− 0.0165	− 0.0003	0.0177	− 0.0199	− 0.0022
	0.975	0.0015	−0.0031	− 0.0016	0.0161	−0.0182	− 0.0021	0.0183	− 0.0224	− 0.0041
200	0.025	−0.0005	− 0.0015	− 0.0020	− 0.0094	0.0090	−0.0004	− 0.0123	0.0098	− 0.0025
	0.05	0.0002	−0.0023	−0.0021	− 0.0086	0.0068	− 0.0018	− 0.0118	0.0093	−0.0025
	0.10	0.0000	0.0016	0.0016	− 0.0095	0.0116	0.0021	−0.0115	0.0134	0.0019
	0.20	0.0023	−0.0002	0.0021	−0.0059	0.0074	0.0015	−0.0072	0.0083	0.0011
	0.80	0.0018	−0.0056	− 0.0038	0.0089	− 0.0122	− 0.0033	0.0106	− 0.0141	− 0.0035
	0.90	−0.0002	0.0002	0.0000	0.0087	−0.0099	− 0.0012	0.0102	− 0.0124	− 0.0022
	0.95	0.0024	−0.0006	0.0018	0.0117	−0.0111	0.0006	0.0131	−0.0137	−0.0006
	0.975	0.0017	−0.0001	0.0016	0.0120	−0.0096	0.0024	0.0131	−0.0127	0.0004

**Table 3:** The error between simulated coverage probability and nominal coverage probability for the 95% two-sided and 97.5% one-sided when *N* = 10, 20, and 30

		Exact approach			Chakraborti and Li [24]			Bland and Altman [2]		
*N*	*p*	Upper	Lower	Two-sided	Upper	Lower	Two-sided	Upper	Lower	Two-sided
		97.5% CI	97.5% CI	95% CI	97.5% CI	97.5% CI	95% CI	97.5% CI	97.5% CI	95% CI
10	0.025	0.0017	0.0010	0.0027	− 0.0350	0.0247	− 0.0103	−0.0464	0.0247	−0.0217
	0.05	0.0012	0.0008	0.0020	−0.0328	0.0246	−0.0082	−0.0440	0.0246	−0.0194
	0.10	−0.0001	0.0016	0.0015	−0.0308	0.0239	−0.0069	−0.0405	0.0239	−0.0166
	0.20	0.0014	−0.0012	0.0002	−0.0231	0.0200	−0.0031	−0.0275	0.0203	−0.0072
	0.80	−0.0002	−0.0009	− 0.0011	0.0192	− 0.0234	−0.0042	0.0195	−0.0288	− 0.0093
	0.90	0.0018	−0.0005	0.0013	0.0231	−0.0282	−0.0051	0.0231	−0.0384	− 0.0153
	0.95	0.0009	0.0011	0.0020	0.0245	−0.0341	−0.0096	0.0245	−0.0481	− 0.0236
	0.975	−0.0015	0.0023	0.0008	0.0246	−0.0335	−0.0089	0.0246	−0.0487	− 0.0241
20	0.025	0.0031	−0.0007	0.0024	−0.0221	0.0215	−0.0006	−0.0296	0.0216	−0.0080
	0.05	0.0027	−0.0006	0.0021	−0.0213	0.0207	−0.0006	−0.0276	0.0213	−0.0063
	0.10	−0.0016	−0.0013	− 0.0029	−0.0237	0.0192	−0.0045	− 0.0294	0.0198	− 0.0096
	0.20	−0.0008	−0.0004	− 0.0012	−0.0166	0.0153	−0.0013	− 0.0208	0.0157	− 0.0051
	0.80	0.0007	0.0016	0.0023	0.0168	−0.0161	0.0007	0.0177	−0.0206	−0.0029
	0.90	0.0020	−0.0002	0.0018	0.0188	−0.0245	−0.0057	0.0189	−0.0300	− 0.0111
	0.95	0.0016	0.0003	0.0019	0.0222	−0.0256	−0.0034	0.0223	−0.0324	− 0.0101
	0.975	−0.0021	−0.0013	− 0.0034	0.0216	− 0.0287	−0.0071	0.0220	−0.0372	− 0.0152
30	0.025	0.0023	−0.0019	0.0004	−0.0215	0.0179	−0.0036	−0.0254	0.0183	−0.0071
	0.05	0.0024	−0.0025	−0.0001	− 0.0200	0.0162	− 0.0038	−0.0245	0.0172	−0.0073
	0.10	−0.0011	0.0014	0.0003	−0.0206	0.0164	−0.0042	−0.0256	0.0170	−0.0086
	0.20	0.0018	−0.0017	0.0001	−0.0131	0.0131	0.0000	−0.0161	0.0136	−0.0025
	0.80	0.0016	0.0001	0.0017	0.0142	−0.0156	−0.0014	0.0150	−0.0196	− 0.0046
	0.90	0.0018	−0.0003	0.0015	0.0166	−0.0205	−0.0039	0.0170	−0.0256	− 0.0086
	0.95	0.0006	0.0014	0.0020	0.0198	−0.0181	0.0017	0.0201	−0.0230	−0.0029
	0.975	−0.0017	0.0004	−0.0013	0.0194	−0.0214	−0.0020	0.0199	−0.0265	− 0.0066

**Table 4:** The error between simulated coverage probability and nominal coverage probability for the 95% two-sided and 97.5% one-sided confidence intervals when *N* = 50, 100, and 200

		Exact approach			Chakraborti and Li [24]			Bland and Altman [2]		
*N*	*p*	Upper	Lower	Two-sided	Upper	Lower	Two-sided	Upper	Lower	Two-sided
		97.5% CI	97.5% CI	95% CI	97.5% CI	97.5% CI	95% CI	97.5% CI	97.5% CI	95% CI
50	0.025	−0.0004	− 0.0022	−0.0026	− 0.0188	0.0144	− 0.0044	−0.0221	0.0157	−0.0064
	0.05	−0.0002	−0.0026	− 0.0028	−0.0169	0.0131	−0.0038	− 0.0208	0.0145	− 0.0063
	0.10	0.0007	−0.0018	−0.0011	− 0.0135	0.0104	− 0.0031	−0.0164	0.0120	−0.0044
	0.20	0.0009	−0.0020	−0.0011	− 0.0083	0.0084	0.0001	−0.0105	0.0089	−0.0016
	0.80	−0.0001	−0.0008	− 0.0009	0.0102	− 0.0114	−0.0012	0.0112	−0.0132	− 0.0020
	0.90	0.0020	−0.0017	0.0003	0.0138	−0.0161	−0.0023	0.0146	−0.0189	− 0.0043
	0.95	0.0031	0.0006	0.0037	0.0159	−0.0145	0.0014	0.0161	−0.0183	−0.0022
	0.975	0.0014	−0.0010	0.0004	0.0160	−0.0182	−0.0022	0.0167	−0.0227	− 0.0060
100	0.025	0.0015	−0.0017	−0.0002	− 0.0096	0.0105	0.0009	−0.0119	0.0116	−0.0003
	0.05	0.0007	−0.0030	−0.0023	− 0.0090	0.0091	0.0001	−0.0108	0.0106	−0.0002
	0.10	0.0002	−0.0008	−0.0006	− 0.0106	0.0095	− 0.0011	−0.0128	0.0101	−0.0027
	0.20	−0.0002	0.0035	0.0033	−0.0090	0.0110	0.0020	−0.0104	0.0115	0.0011
	0.80	0.0014	0.0008	0.0022	0.0088	−0.0075	0.0013	0.0092	−0.0094	−0.0002
	0.90	0.0005	0.0000	0.0005	0.0111	−0.0096	0.0015	0.0121	−0.0120	0.0001
	0.95	0.0013	−0.0012	0.0001	0.0115	−0.0143	−0.0028	0.0117	−0.0164	− 0.0047
	0.975	0.0014	−0.0011	0.0003	0.0118	−0.0140	−0.0022	0.0125	−0.0160	− 0.0035
200	0.025	0.0006	−0.0019	−0.0013	− 0.0066	0.0077	0.0011	−0.0084	0.0084	0.0000
	0.05	0.0021	−0.0013	0.0008	−0.0056	0.0075	0.0019	−0.0077	0.0079	0.0002
	0.10	0.0005	0.0023	0.0028	−0.0068	0.0088	0.0020	−0.0079	0.0095	0.0016
	0.20	0.0012	−0.0012	0.0000	−0.0042	0.0047	0.0005	−0.0052	0.0053	0.0001
	0.80	0.0029	−0.0017	0.0012	0.0077	−0.0077	0.0000	0.0080	−0.0095	−0.0015
	0.90	−0.0015	−0.0001	− 0.0016	0.0054	− 0.0070	−0.0016	0.0061	−0.0085	− 0.0024
	0.95	0.0020	−0.0021	−0.0001	0.0100	−0.0089	0.0011	0.0104	−0.0100	0.0004
	0.975	0.0016	0.0003	0.0019	0.0093	−0.0080	0.0013	0.0103	−0.0099	0.0004

**Fig. 1:**
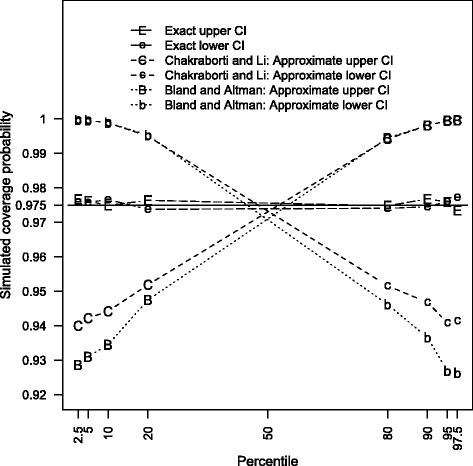
Coverage probability of 97.5% one-sided confidence interval for *N* = 10

It can be seen from the resulting errors of the three types of confidence intervals that the exact approach performs extremely well for all 96 cases presented in Tables 1, 2, 3 and 4. For the two approximate methods of Chakraborti and Li [24] and Bland and Altman [2], the coverage probabilities of their two-sided interval remain rather close to the nominal confidence levels. However, the corresponding approximate one-sided interval procedures do not preserve the same desired accuracy unless the sample size is large. Due to different degree of presumed simplifications, the interval procedure of Bland and Altman [2] is inferior to that of Chakraborti and Li [24], especially for small sample sizes. To enhance the explication, the simulated coverage probabilities of the 97.5% one-sided confidence intervals for *N* = 10 are plotted in Fig. 1. Despite the attractive coverage behavior of the approximate two-sided confidence intervals, the errors of the upper confidence intervals tend to be negative for small *p* while those associated with large *p* are consistently positive. The situations of the lower confidence intervals reveal exactly the opposite patterns. In other words, the corresponding lower and upper confidence limits are generally too large for the 2.5th, 5th, 10th and 20th normal percentiles and are mostly too small for the 80th, 90th, 95th, and 97.5th normal percentiles. Consequently, the two endpoints of the two-sided confidence intervals generally do not meet the assumption of equal-tailed error rates for the two approximate interval methods. A mere coverage probability assessment of the approximate two-sided confidence intervals may obscure the potential biases of the confidence limits based on the *t*(ν) approximations described in Eqs. 11 and 13. It is inappropriate to claim that a two-sided interval procedure is accurate on the basis of a combination of some noticeable under- and over-estimated confidence limits. Instead, the exact interval procedure should be used in preference to the approximate methods of Bland and Altman [2] and Chakraborti and Li [24].

### Sample size determinations

From a study design viewpoint, it is essential to determine the optimal sample sizes so that the resulting confidence interval will meet the designated precision requirement. Two particularly useful criteria concern the control of the expected width and the assurance probability of the width within a designated bound (Beal [26]; Kupper & Hafner [27]).

The width of the 100(1 – α)% two-sided confidence intervals {$\widehat{\uptheta}$_*L*_, $\widehat{\uptheta}$_*U*_} given in Eq. 7 is

17 $$ W=\left\{{t}_{1-\upalpha /2}\left(v,{z}_p{N}^{1/2}\right)-{t}_{\upalpha /2}\left(v,{z}_p{N}^{1/2}\right)\right\}\left(S/{N}^{1/2}\right). $$

Accordingly, it is desired to calculate the least sample size such that the expected width of a 100(1 – α)% two-sided confidence interval is within the given threshold:

18 $$ E\left[W\right]\le \updelta, $$

where δ (> 0) is a constant. On the other hand, one may compute the minimum sample size needed to guarantee, with a given assurance probability, that the width of a 100(1 – α)% two-sided confidence interval will not exceed the planned value:

19 $$ P\left(W\le \upomega \right)\ge 1-\upgamma, $$

where 1 – γ is the specified assurance level and ω (> 0) is a constant.

Under the normal assumption, the assessments of expected width and assurance probability are further simplified for brevity. Note that the expected width *E*[*W*] has the alternative form

20 $$ E\left[W\right]=\left\{{t}_{1-\upalpha /2}\left(v,{z}_p{N}^{1/2}\right)-{t}_{\upalpha /2}\left(v,{z}_p{N}^{1/2}\right)\right\}\left\{\upsigma /\left({cN}^{1/2}\right)\right\}. $$

Hence, the inequality *E*[*W*] ≤ δ is expressed as {*t*_1 − α/2_(ν, *z*_*p*_*N*^1/2^) – *t*_α/2_(ν, *z*_*p*_*N*^1/2^)}/(*cN*^1/2^) ≤ δ/σ. Also, the assurance probability is equivalent to

21 $$ P\left(W\le \upomega \right)=P\left(K<\upkappa \right)=\Phi \left(\upkappa \right), $$

where *K* = ν*S*^2^/σ^2^ ~ χ^2^(ν) is a chi-square distribution with ν degrees of freedom, κ = {*N*(*N* – 1)(ω/σ)^2^}/{*t*_1 − α/2_(ν, *z*_*p*_*N*^1/2^) – *t*_α/2_(ν, *z*_*p*_*N*^1/2^)}^2^, and Φ(*·*) is the cumulative distribution function of the chi-square random variable *K*. With the exact computational formulas of expected width and assurance probability given in Eqs. 20 and 21, respectively, the sample size *N* needed to attain the specified precision can be found with a simple iterative search for the chosen parameter values {μ, σ^2^}, percentile *p*, and confidence level 1 – α.

Evidently, the sample size determinations do not depend on the mean value μ and reduce to the sample size procedures of Kupper and Hafner [27] because θ = μ when *p* = 0.5. The precision evaluations of expected width and assurance probability depend on the thresholds δ and ω through the relative magnitude ratios δ/σ and ω/σ, respectively. Accordingly, supplementary SAS/IML and R computer programs are presented to facilitate the required computations. Due to the prospective nature of advance research planning, the general guidelines suggest that typical sources like published findings or expert opinions can offer plausible and reasonable values for the vital characteristics of future study. For illustration, the sample statistics of the blood pressure data in Bland and Altman [2] are adopted as parameter values μ = − 16.29 and σ = 19.61. With δ = ω = (0.7)σ = 9.805 and 1 – γ = 0.9, the optimal sample sizes for precise 95% interval estimation of the 97.5th percentile are 183 and 207 under the expected width and assurance probability criteria, respectively. For ease of application, the prescribed configurations are incorporated in the user specification sections of the SAS/IML (Additional files 1, 2 and 3) and R programs (Additional files 4, 5 and 6).

**Fig. 2:**
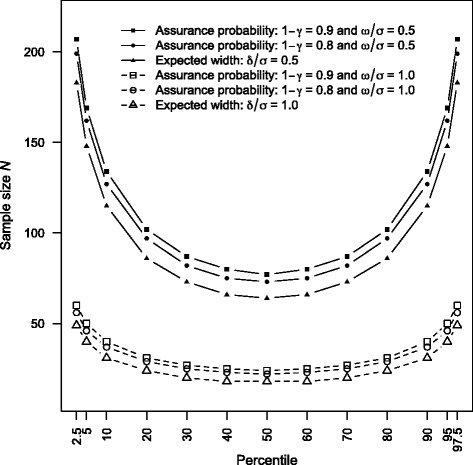
Computed sample size for precise interval estimation of percentile

To further demonstrate the features and differences of the two suggested sample size procedures for precise interval estimation of the normal percentiles, numerical computations are performed for *p* = 0.025, 0.05, 0.10, 0.20, 0.30, 0.40, 0.50, 0.60, 0.70, 0.80, 0.90, 0.95, and 0.975 under the expected width and assurance probability criteria. The parameter configurations are fixed as μ = 0, σ^2^ = 1, 1 – α = 0.95 throughout the empirical appraisal. Moreover, the selected two thresholds of expected width are δ = 0.5 and 1.0. For assurance evaluation, the four designated settings are 1 – γ = 0.80 and 0.9 combined with ω = 0.5 and 1.0. These configurations are chosen to reflect common sample sizes used in typical research settings. For ease of illustration, the computed sample sizes are plotted in Fig. 2.

It is seen from Fig. 2 for the six types of precision that the graphs of the optimal sample size are symmetric with respect to *p* = 0.5 and are monotonously increasing with the absolute difference |*p* – 0.5|. Therefore, the required sample size for precise interval estimation of median or mean is smaller than those of the other normal percentiles. Also, the optimal sample size increases with a smaller width bound of δ and ω when all other factors are fixed. As expected, more sample size is needed to attain a higher assurance level 1 – γ when the designated width ω and other configurations remain identical. Regarding the difference between the two precision principles, it typically requires a larger sample size to meet the necessary precision of assurance probability than the control of a designated expected width. With the same interval bound δ = ω, the sample sizes associated with the assurance criterion are larger than those under the expected width consideration. For the precision settings considered here, the sample sizes for δ/σ = ω/σ = 1.0 are within the range of [40, 60] for *p* = 0.95 and 0.975. With δ/σ = ω/σ = 0.5, the computed sample sizes for the same percentiles are much larger and have a wider interval [148, 207]. These numerical illustrations suggest that the width bounds δ/σ = ω/σ = 0.5 and 1.0 and the assurance level 1 – γ = 0.80 and 0.90 lead to sensible sample sizes and are suitable benchmark precision setups for designing percentile studies. Deciding on the appropriate precision requirements always requires careful thought and should be determined by the research context and study goal within a particular scientific field.

## Discussion

In view of the wide application in medical studies, this article aims to explicate the theoretical and empirical features of interval procedures of percentiles. An integrated discussion is presented to address the similarities and differences of exact and approximate confidence intervals constructed with various pivotal quantities described in the literature. Although there are distinct selections of pivotal quantities, it is shown that they yield the same exact confidence intervals. Notably, the exact interval procedure requires the use of the cumulative distribution function of a noncentral *t* distribution. The difficulty of applying the exact approach has been alleviated because of the availability of specialized routines in popular software packages. In contrast, the approximate interval methods are computationally simple and do not require specialized software because they only involve the quantiles of a regular *t* distribution. However, the approximate confidence intervals carry the symmetry property of a *t* distribution whereas the noncentral *t* distribution is skewed so that the resulting exact confidence intervals are not equidistant around the primary statistic.

## Conclusions

Despite the positive findings in previous research, detailed numerical assessments are presented to reveal the underlying drawbacks of the approximate methods under the notion that the endpoints of a two-sided confidence interval have a corresponding interpretation as a lower or upper confidence limit of a one-sided confidence interval. Essentially, the simplicity and symmetry of an approximate confidence interval generally do not maintain the assumption of equal-tailed error rates for the two individual endpoints. For the purpose of planning percentile studies so that the results will help confirm meaningful reference targets, sample size procedures for precise interval estimation of normal percentiles are described under the precision criteria of expected width and assurance probability. To enhance the applicability of the exact interval approach and corresponding sample size methodologies, computer codes are also presented to perform the required computations.

## Additional files

Additional file 1: SAS/IML program for computing the exact confidence interval of percentile. (DOCX 64 kb)

Additional file 2: SAS/IML program for computing sample size required to meet the designated expected width for confidence interval of percentile. (DOCX 64 kb)

Additional file 3: SAS/IML program for computing sample size required to ensure adequate assurance probability of achieving the desired width for confidence interval of percentile. (DOCX 67 kb)

Additional file 4: R program for computing the exact confidence interval of percentile. (DOCX 62 kb)

Additional file 5: R program for computing sample size required to meet the designated expected width for confidence interval of percentile. (DOCX 64 kb)

Additional file 6: R program for computing sample size required to ensure adequate assurance probability of achieving the desired width for confidence interval of percentile. (DOCX 20 kb)
