# Supplementary material for: The appropriateness of Bland-Altman’s approximate confidence intervals for limits of agreement
Source: BMC Med Res Methodol. 2018 May 22;18:45. doi: 10.1186/s12874-018-0505-y (PMC5964973; doi:10.1186/s12874-018-0505-y)
Supplement: Supplementary file 1 — SAS/IML program for computing the exact confidence interval of percentile. (DOCX 64 kb) [file 12874_2018_505_MOESM1_ESM.docx]

Additional file 1

SAS/IML program for computing the exact confidence interval of percentile

PROC IML;

*USER SPECIFICATION PORTION;

*DESIGNATED ALPHA;ALPHA=0.05;

*SAMPLE SIZE;N=85;

*SAMPLE MEAN;MEAN=-16.29;

*SAMPLE STANDARD DEVIATION;S=19.61;

*PERCENTILE;PCT=0.975;

*END OF USER SPECIFICATION PORTION;

ZP=QUANTILE('NORMAL',PCT);

COVERP=1-ALPHA

DF=N-1;

LOGC=LOG(SQRT(DF/2))+LGAMMA(DF/2)-LGAMMA(N/2);C=EXP(LOGC);

THETAH=MEAN+ZP#C#S;

VARH=(S#S/N)#(1+N#ZP#ZP#(C#C-1));

STDH=SQRT(VARH);

TL=QUANTILE('T',ALPHA/2,DF,ZP#SQRT(N));

TU=QUANTILE('T',1-ALPHA/2,DF,ZP#SQRT(N));

CL=MEAN+TL#S/SQRT(N);

CU=MEAN+TU#S/SQRT(N);

PRINT ALPHA COVERP N;

PRINT MEAN S[FORMAT=8.4] PCT ZP[FORMAT=8.4];

PRINT THETAH[FORMAT=8.4] VARH[FORMAT=8.4] STDH[FORMAT=8.4];

PRINT 'EXACT CI' CL[FORMAT=8.4] CU[FORMAT=8.4];

QUIT;
