# Supplementary material for: The appropriateness of Bland-Altman’s approximate confidence intervals for limits of agreement
Source: BMC Med Res Methodol. 2018 May 22;18:45. doi: 10.1186/s12874-018-0505-y (PMC5964973; doi:10.1186/s12874-018-0505-y)
Supplement: Supplementary file 2 — SAS/IML program for computing sample size required to meet the designated expected width for confidence interval of percentile. (DOCX 64 kb) [file 12874_2018_505_MOESM2_ESM.docx]

Additional file 2

SAS/IML program for computing sample size required to meet the designated expected width for confidence interval of percentile

PROC IML;

*USER SPECIFICATION PORTION;

*DESIGNATED ALPHA;ALPHA=0.05;

*MEAN;MU=-16.29;

*STANDARD DEVIATION;SIGMA=19.61;

*PERCENTILE;PCT=0.975;

*EXPECTED WIDTH;EW=0.5#SIGMA;

*END OF USER SPECIFICATION PORTION;

ZP=QUANTILE('NORMAL',PCT);

SIGSQ=SIGMA##2;THETA=MU+ZP#SIGMA;

COVERP=1-ALPHA;

PRINT ALPHA COVERP EW;

PRINT MU SIGMA[FORMAT=8.4] PCT ZP[FORMAT=8.4] THETA[FORMAT=8.4];

N=4;

DO UNTIL(EWE<EW);

N=N+1;DF=N-1;

LOGC=LOG(SQRT(DF/2))+LGAMMA(DF/2)-LGAMMA(N/2);C=EXP(LOGC);

TL=QUANTILE('T',ALPHA/2,DF,ZP#SQRT(N));

TU=QUANTILE('T',1-ALPHA/2,DF,ZP#SQRT(N));

TD=TU-TL;

EWE=TD#SIGMA/(C#SQRT(N));

END;

PRINT EW EWE[FORMAT=8.4] N;

QUIT;
