# Supplementary material for: The appropriateness of Bland-Altman’s approximate confidence intervals for limits of agreement
Source: BMC Med Res Methodol. 2018 May 22;18:45. doi: 10.1186/s12874-018-0505-y (PMC5964973; doi:10.1186/s12874-018-0505-y)
Supplement: Supplementary file 4 — R program for computing the exact confidence interval of percentile. (DOCX 62 kb) [file 12874_2018_505_MOESM4_ESM.docx]

Additional file 4

R program for computing the exact confidence interval of percentile

function () {

#USER SPECIFICATIONS PORTION

alpha<-0.05 #DESIGNATED ALPHA

n<-85 #SAMPLE SIZE

mean<--16.29 #SAMPLE MEAN

s<-19.61 #SAMPLE STANDARD DEVIATION

pct<-0.975 #PERCENTILE

#END OF SPECIFICATION

zp<-qnorm(pct)

coverp<-1-alpha

df<-n-1

logc<-log(sqrt(df/2))+lgamma(df/2)-lgamma(n/2)

c<-exp(logc)

thetah<-mean+zp*c*s

varh<-(s^2/n)*(1+n*zp^2*(c*(c-1)))

stdh<-sqrt(varh)

tl<-qt(alpha/2,df,zp*sqrt(n))

tu<-qt(1-alpha/2,df,zp*sqrt(n))

cl<-mean+tl*s/sqrt(n)

cu<-mean+tu*s/sqrt(n)

return(list(alpha=alpha,coverp=coverp,n=n,mean=mean,s=s,pct=pct,zp=zp,thetah=thetah,varh=varh,stdh=stdh,'exact ci',cl=cl,cu=cu))

}
