# Supplementary material for: The appropriateness of Bland-Altman’s approximate confidence intervals for limits of agreement
Source: BMC Med Res Methodol. 2018 May 22;18:45. doi: 10.1186/s12874-018-0505-y (PMC5964973; doi:10.1186/s12874-018-0505-y)
Supplement: Supplementary file 5 — R program for computing sample size required to meet the designated expected width for confidence interval of percentile. (DOCX 64 kb) [file 12874_2018_505_MOESM5_ESM.docx]

Additional file 5

R program for computing sample size required to meet the designated expected width for confidence interval of percentile

function () {

#USER SPECIFICATIONS PORTION

alpha<-0.05 #DESIGNATED ALPHA

mu<--16.29 #MEAN

sigma<-19.61 #STANDARD DEVIATION

pct<-0.975 #PERCENTILE

ew<-0.5*sigma #EXPECTED WIDTH

#END OF SPECIFICATION

zp<-qnorm(pct)

sigsq<-sigma^2

theta<-mu+zp*sigma

coverp<-1-alpha

n<-4

ewe<-100

while(ewe>ew){

n<-n+1

df<-n-1

logc<-log(sqrt(df/2))+lgamma(df/2)-lgamma(n/2)

c<-exp(logc)

tl<-qt(alpha/2,df,zp*sqrt(n))

tu<-qt(1-alpha/2,df,zp*sqrt(n))

td<-tu-tl

ewe<-td*sigma/(c*sqrt(n))

}

return(list(alpha=alpha,coverp=coverp,ew=ew,mu=mu,sigma=sigma,pct=pct,zp=zp,theta=theta,ewe=ewe,n=n))

}
